# Implementation of green-assessed nanotechnology and quality by design approach for development of optical sensor for determination of tobramycin in ophthalmic formulations and spiked human plasma

**DOI:** 10.1186/s13065-024-01234-y

**Published:** 2024-07-15

**Authors:** Christine M. El-Maraghy, Passant M. Medhat, Rania M. Hathout, Miriam F. Ayad, Nermine V. Fares

**Affiliations:** 1 Analytical Chemistry Department, Faculty of Pharmacy, October University for Modern Sciences and Arts (MSA), 6th October City, 11787 Cairo Egypt; 2 Pharmaceutics and Industrial Pharmacy Department, Faculty of Pharmacy, Ain Shams University, Abbassia, Cairo, 11566 Egypt; 3 Pharmaceutical Analytical Chemistry Department, Faculty of Pharmacy, Ain Shams University, Abbassia, Cairo, 11566 Egypt

**Keywords:** Quality by design, Colorimetry, Sensor, Tobramycin, Green, Silver nanoparticles, AGREE, GAPI

## Abstract

A fast eco-friendly colorimetric method was developed for the determination of Tobramycin in drug substance, ophthalmic formulations, and spiked human plasma using silver nanoparticles optical sensor. Even though tobramycin is non-UV–visible absorbing, the developed method is based on measuring the absorbance quenching of silver nanoparticles resulting from the interaction with tobramycin. Different factors affecting the absorbance intensity were studied as; silver nanoparticle concentration, pH, buffer type, and reaction time using quality by design approach. Validation of the proposed method was performed according to ICH guidelines and was found to be accurate, precise, and sensitive. The linearity range of tobramycin was 0.35–4.0 μg/mL. The optical sensor was successfully applied for the determination of Tobramycin in ophthalmic formulations and spiked human plasma without pre-treatment. Additionally, the binding between Tobramycin and PVP- capped silver nanoparticles was studied using molecular docking software. The method was assessed and compared to colorimetric reported methods for the green character using Green Analytical Procedure Index (GAPI) and Analytical GREEnness calculator (AGREE) tools and found to be greener.

**Supplementary Information:**

The online version contains supplementary material available at 10.1186/s13065-024-01234-y.

## Introduction

Tobramycin (TOBRA), Supplementary material Fig. S1, is an aminoglycoside produced by *Streptomyces Tenebrarius* [1]. It is effective against gram-positive and gram-negative bacteria. TOBRA was widely used during COVID-19 pandemic to treat the secondary bacterial infection caused by viral pneumonia [2]. Moreover, it was used to treat symptoms of conjunctivitis frequently associated with COVID-19 patients [3]. The literature reveals some spectroscopic [4, 5], and chromatographic [6, 7] methods for the determination of TOBRA but they had the disadvantages of using the derivatization step which is time-consuming, tedious, and not eco-friendly in addition, it lowers the method sensitivity. The analysis of TOBRA is challenging as it has no absorbance in the UV/visible region, and it is very polar so has weak retention on the reversed-phase LC. For those mentioned reasons, we aimed to develop a rapid green visual colorimetric method for the analysis of TOBRA, which does not depend on its absorption nor utilize the derivatization process. Consequently, the absorbance quenching method using nanoparticles was applied to achieve this goal. The principle of the absorbance quenching method depends on decreasing the absorbance intensity of silver nanoparticles (AgNps) upon reaction with TOBRA through hydrogen bond formation, resulting in aggregation of AgNps [8, 9], and hence, a color change occurred which could be measured quantitatively. We chose the (AgNps) as they are inexpensive and have high extinction for colorimetric detection [10], special optical and electronic properties [11], and they are widely used in bio-sensing, photonics, and in antimicrobial applications as the manufacturing of biodegradable surgical sutures [12]. Additionally, new insights on the application of AgNps were applied in drug delivery[13], glucose determination [14], DNA detection [15], analysis of drugs [16–18], heavy metal ions [19, 20], anions [10, 21], and cations [22].

**Fig. 1:**
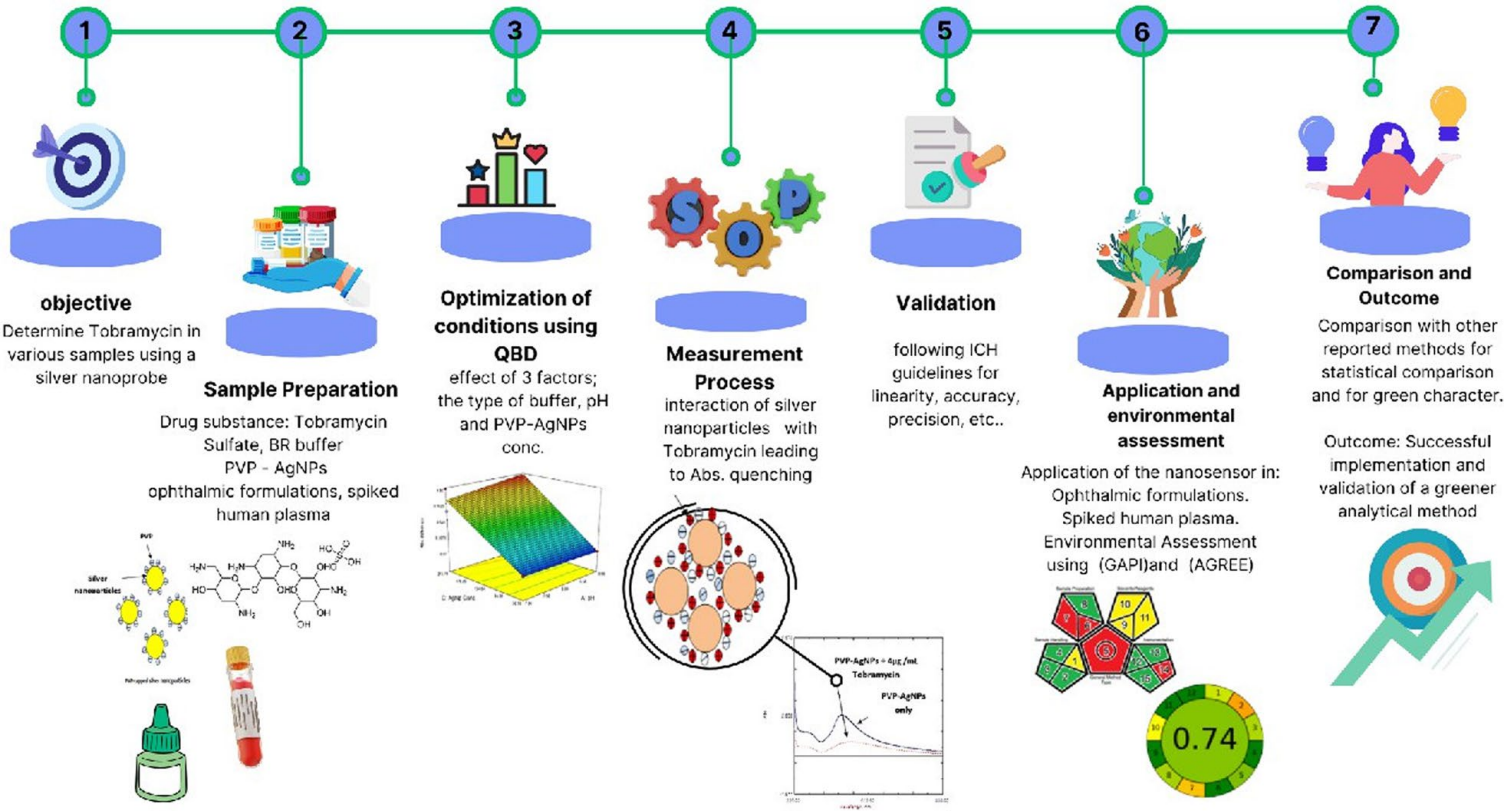
A flow diagram that describes the steps of the proposed method

Few colorimetric methods were reported for the determination of TOBRA using sodium dodecyl sulfate (SDS) capped -AgNps in milk and in exhaled breath condensate (EBC) [23–25], and using citrate capped -AgNp decorated with TOBRA-specific aptamers for analysis of TOBRA in milk [26], the comparison between the reported and proposed methods is shown in Supplementary material Table S1. In this work, the type of stabilizer used for capping AgNPs is polyvinyl pyrrolidone (PVP); as they have long polyvinyl chains that act as steric protective shields on the AgNps surface which make them stable and dispersed when compared to the SDS and citrate capping agent [27]. Moreover, neither of the published methods applied the quality-by-design (QbD) approach nor implemented the green chemistry principles for the colorimetric analysis of TOBRA in pharmaceutical preparation and human plasma. The (QbD) approach enables us to choose the best reaction conditions using minimal number of trials to achieve maximum absorbance quenching and hence the optimum method performance. Furthermore, none of these methods determined TOBRA in biological fluid. The green character of the developed method was assessed and compared to colorimetric reported methods by Green Analytical Procedure Index (GAPI) [28] and Analytical GREEnness calculator (AGREE) tools [29]. Additionally, the interaction between the TOBRA and PVP capped AgNPs (PVP-AgNPs) was assessed using molecular docking software. Thus, in this work, a fast, eco-friendly, and validated colorimetric method was developed for determination of the non-chromophoric TOBRA based on absorbance quenching of PVP-AgNPs in ophthalmic formulations and spiked human plasma. Figure 1 shows a flow diagram that summarizes the steps of the proposed method.

## Experimental

### Materials and reagents

TOBRA (purity: 100.74% ± 0.5), Al Andalous for pharmaceutical industries (Giza, Egypt), its purity was checked by the colorimetric method [4]. PVP-AgNPs dark yellow, water-soluble colloidal solution (particle size 40 nm ± 5 nm) (Nanotech, Giza, Egypt). Orthophosphoric acid, acetic acid, and sodium hydroxide (LobaChemie, India). Boric acid, sodium carbonate, and sodium bicarbonate (Oxford Laboratory reagent, India). Chloroform (Carlo Erba reagents). Bi-distilled water was used throughout the work. Human plasma was obtained from (VACSERA, Cairo, Egypt) and stored at − 20 °C until use.

### Instrumentation

A double beam spectrophotometer (UV-1800, Shimadzu-Japan) connected to a PC with UV-probe 2.10 software. Transmission electron microscopy (TEM) (JOEL JEM-1400, USA). Sigma Centrifuge (model no. D-37520, Germany). Jenway pH glass electrode (Model P14/BNC, Stone, UK).

### Software

Design expert® software version 7.0 was used for experimental design. MOE® version 2014.0901 (Chemical Computing Group Inc., Montreal, Canada) was utilized in docking study. ChemDraw® Ultra version 10 (Cambridgesoft, Waltham, MA) and Chem3D® Ultra version 10 (Cambridgesoft, Waltham, MA) were utilized for drawing the chemical structures.

### Pharmaceutical formulations

Tobrin® ophthalmic solution (batch No. 1801933), 3000 μg TOBRA per 1 mL, EIPICO company and Tobrin® ophthalmic ointment (batch No. 2010036), 3000 μg TOBRA per 1 gm, EIPICO company.

### Buffers preparation

Britton-Robinson buffer was prepared by mixing equal volumes of acetic acid, boric acid and, phosphoric acid adjusted to the required pH by 0.2 M NaOH [30]. Carbonate buffer was prepared by mixing 0.1 M sodium carbonate and 0.1 M sodium bicarbonate, adjusted to the required pH by 0.2 M HCL [31].

### Standard and working solutions

Stock solution of TOBRA was prepared (1000 μg/mL) using bi-distilled water. The working solution was prepared by dilution from the stock to reach a concentration of (50 μg/mL).

### Procedure

#### Absorbance quenching produced by TOBRA-PVP-AgNPs reaction

A solution of PVP-AgNPs of concentration (215.74 µg/mL) was prepared using a solvent mixture of Britton-Robinson buffer (pH 9) and bi-distilled water in a ratio (50:50 v/v) and its absorption spectrum was recorded at λ_max_ = 415 nm using the same solvent mixture as a blank. An aliquot of TOBRA was added to the previously prepared solution of PVP-AgNPs to obtain a final concentration of 4 µg/mL TOBRA then the solution was left to stand for 10 min and the absorbance was measured at 415 nm. The reaction between the TOBRA and the PVP-AgNPs resulted in a color change from yellowish-brown to orange.

### Exploring the mechanism of tobramycin absorbance quenching due to the used PVP-capped silver nanoparticles using molecular docking studies

#### Building PVP polymeric matrix

The PVP polymeric matrix constituting of 5 or 10 monomers was constructed from the polymer SMILES;CC(N1CCCC1(=O))CC(N1CCCC1(=O))CC(N1CCCC1(=O))CC(N1CCCC1(= O))CC(N1CCCC1(=O))CC(N1CCCC1(=O))CC(N1CCCC1(=O))CC(N1CCCC1(=O))CC(N1CCCC1(=O))CC(N1CCCC1(=O))CC, utilizing the chemical sketch tool of the RSCB protein data bank (RCSB PDB: Chemical Sketch Tool).

After, the generated pdb files of the investigated polymer was imported to MOE® version 2014.0901, where the energy minimization of PVP chemical structure was performed using the MMFF94x forcefield simulation tool found in the MOE software package [32].

### Construction of the chemical structure of tobramycin and its preparation for docking

The chemical structure of TOBRA was built using ChemDraw® Ultra version 10 from its isomeric SMILES that were obtained from PubChem [33]. The corresponding Mol2 file (usually needed for docking experiments) was obtained using Chem3D® Ultra version 10. The energy minimization to obtain the most stable 3D configuration in space was performed utilizing the MM2 forcefield of the same software [34].

### Molecular docking studies

The molecular docking analysis was adopted using MOE® version 2014.0901. Binding sites identification was carried out using the MOE's "Site finder" tool [35]**.** The "*triangle matcher”* as the placement method in the docking technique [36] and the London score of the software was exploited to generate the free binding energies results of the docking process.

### Validation of the proposed method

The analytical method validation of the proposed method was done according to International Council on Harmonization recommendations [37]. For the linearity, aliquots of TOBRA were added to the solution of PVP-AgNPs to obtain concentrations range (0.35–4.0 μg/mL) then the same procedure was followed. Calibration curve was constructed by plotting (A⁰- A) versus the corresponding concentration of TOBRA and the regression equation was calculated. Accuracy was assessed by measuring the average recoveries and SD of four concentrations of TOBRA (0.6, 0.7, 2.0 and 2.5 μg/mL) using the corresponding regression equation. The intra-day precision (conducted in the same day) and inter-day precision (conducted in three successive days) were determined by measuring three concentrations of TOBRA (1, 2.5, and 3.0 μg/mL) and the relative standard deviation was calculated for each concentration. Approaches based on the SD of the intercept and the slope were used for determining the LOD and LOQ, where LOD = 3.3 × SD/slope and LOQ = 10 × SD/slope.

### Application to pharmaceutical preparations

#### Ophthalmic solution

1 mL of Tobrin® ophthalmic solution was transferred into a 100 mL-volumetric flask and then the volume was completed with bi-distilled water (stock 1). Then, 150 μL were transferred from (stock 1) into a 10 mL-volumetric flask to obtain a final concentration of 0.45 μg/mL of TOBRA. The absorbance quenching of PVP-AgNPs after the addition of the prepared TOBRA solution was measured. The recovery was obtained from the regression equation. The standard addition technique was applied by adding different known concentrations of TOBRA to a known concentration of Tobrin® preparation.

#### Ophthalmic ointment

0.06 gm of Tobrin® ophthalmic ointment was accurately transferred into a separating funnel, dissolved in 1.5 mL chloroform, shaken vigorously for 1 min then extracted with 3 cycles of 10 mL water. The three aqueous extracts were mixed and diluted with bi-distilled water to reach the final concentration of 1μg/mL TOBRA. Then the same procedure was followed, and the standard addition technique was applied.

### Application to human plasma

The ophthalmic route of TOBRA has some systemic absorption; the mean peak serum level reaches 5 µg/mL [38] and consequently, it may cause side effects such as headache and laryngospasm [39]. Therefore, a fast and sensitive method is required for its determination in Human plasma. 1 mL plasma was spiked with different aliquots of TOBRA transferred from the working solution and 2 mL of methanol was added for extraction. The samples were mixed well and centrifuged at 8000 rpm for 10 min. The supernatant was transferred into three 10 mL-volumetric flasks to prepare (0.5, 1, and 5 µg/mL) TOBRA solutions, then analyzed as described.

## Results and discussion

Being a non-chromophoric drug, the spectrophotometric analysis of TOBRA is challenging. Spectrophotometric analysis has many advantages, as it is a time and cost-saving, straightforward technique, which does not require a large volume of solvent so it is considered a green alternative to the commonly applied chromatographic methods. This work applied the absorbance quenching technique using PVP-AgNPs to measure the TOBRA concentration at a specific wavelength. There are two published works for the colorimetric analysis of TOBRA [4, 5]. Both methods have higher LOQ than the proposed method (500 µg/mL and 3 µg/mL, respectively versus 0.3 µg/mL) in addition they are less accurate and less precise which is due to the derivatization step by the diphenylamine reagent in the first method and vanillin in the second method while applying high temperature (around 100 °C). Moreover, neither of the published methods applied the quality-by-design approach nor implemented the green chemistry principle for the spectrophotometric analysis of TOBRA in pharmaceutical preparation and Human plasma, a comparison between the two colorimetric published methods and the proposed method is shown in Supplementary material Table S2.

### Characterization of PVP-AgNPs

**Fig. 2:**
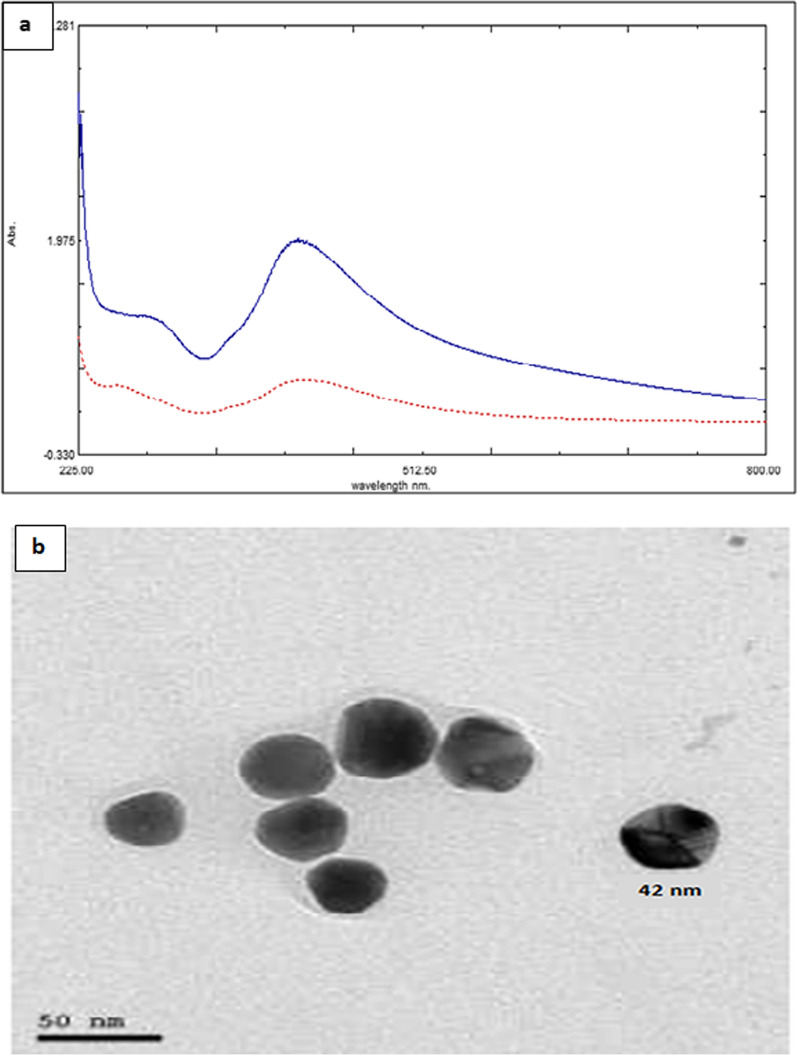
**a** UV–visible absorption spectrum of 215.74 μg/mL PVP-AgNPs (--------) and of PVP-AgNPs reaction with 4 μg/mL TOBRA (…………………) **b** TEM image of PVP-AgNPs

A solution of PVP-AgNPs was characterized by UV spectrophotometer and TEM. The spectrum showed that PVP-AgNPs have SPR peak at 415 nm, which is characteristic for the silver nanoparticles [40, 41] (Fig. 2a). TEM image showed that PVP-AgNPs are spherical in shape, dispersed, and uniform in aqueous solution with particle size 40 nm ± 5 nm (Fig. 2b).

### The mechanism of absorbance quenching of PVP-AgNPs

PVP-AgNPs form a yellow colloidal solution due to excitation of surface Plasmon resonance (SPR) which gives maximum absorption at 415 nm. SPR is the collective oscillation of the conduction electrons on the metal surface when excited by a light of a specific wavelength [40]. The mechanism of PVP-AgNPs aggregation is due to hydrogen bond formation [8] between the lone pair of electrons of the oxygen in the polyvinyl groups of the PVP-AgNPs and hydroxyl and amino groups of TOBRA, Supplementary material Fig. S2. The approach is based on the interaction between opposite charges of AgNPs and TOBRA as the amino and hydroxyl functional groups of TOBRA work as molecular bridge, starting the electrostatic coupling with the neighboring AgNPs. Consequently, an aggregation of the AgNPs occurs and the SPR band of the AgNPs undergoes a hypochromic shift leading to a visible color change from yellowish-brown to orange [9]. Thus, the proposed method is based on measuring the decrease in SPR peak of PVP-AgNPs at 415 nm by increasing the concentration of TOBRA, (Fig. 2a) and hence the absorbance difference (absorbance quenching) between the PVP-AgNPs solution and the TOBRA- PVP-AgNPs solution was measured.

### Probing the tobramycin absorbance quenching mechanism due to the used PVP-capped silver nanoparticles using molecular docking studies

**Fig. 3:**
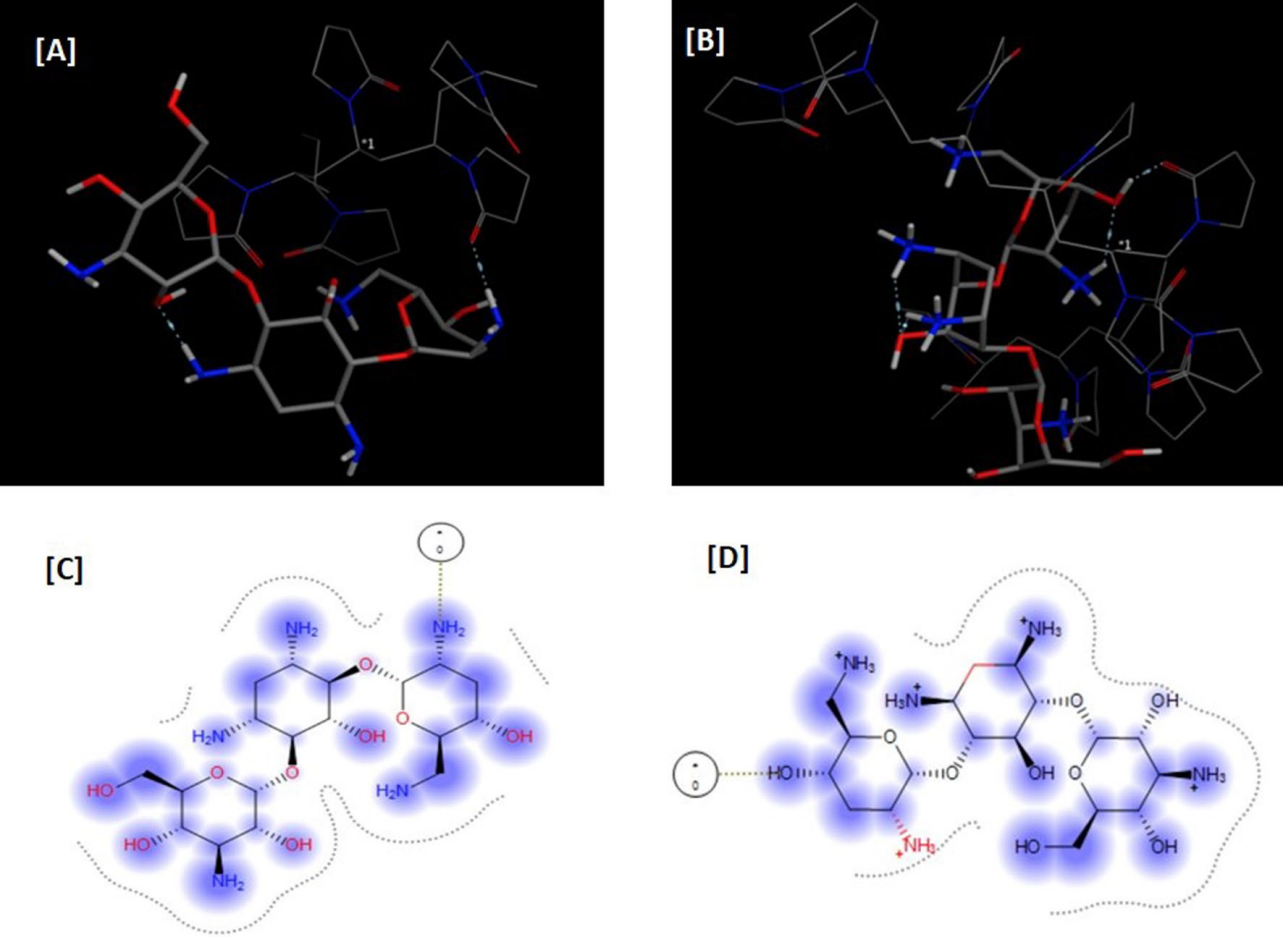
Molecular docking experiments of TOBRA on the capping agent; PVP: **A** and **B** represents the 3D docking results on the 5 and 10 monomeric PVP matrix, respectively, while **C** and **D** represents the corresponding 2D images of the above panel. Tobramycin molecule is presented with balls and sticks while the PVP matrix is presented with wires

Based on the molecular docking studies, TOBRA was found to successfully interact with PVP (ΔG =− 10.23 and − 8.19 kCal for the simulated 5-monomer and 10-monomer PVP, respectively). This was attributed to hydrogen-bond formation between the lone pair of electrons of the oxygen in the polyvinyl groups of the PVP and hydroxyl and amino groups of TOBRA, as demonstrated in Fig. 3

### Preliminary studies

#### Effect of pH on the absorbance quenching

To select the pH for optimum sensitivity of TOBRA with PVP-AgNPs, Britton -Robinson buffer solutions having different pH ranges (5–11) were prepared and tried. The pH range (7–9) was selected for further optimization by quality by design approach. This is because at pH ≤ 4, the moderately increased H^+^ allows the formation of “bridges” between pyrrolidone groups and consequently contracting the AgNPs capping or possibly interconnecting adjacent AgNPs resulting in an increased aggregation rate with higher Zeta potential values [42]. This may also be due to the neutralization of the PVP-AgNPs surface charges and/or absence/reduction of electrostatic repulsion ultimately leading to an increase in the destabilization of the particles and increased particle aggregation [43, 44]. An Increase in pH (from 7 to 9) leads to an increase in the concentration of OH^−^ thus increasing electrostatic repulsion of PVP-AgNPs and thereby providing stabilization at alkaline pH which is translated as decreased particle size with small hydrodynamic diameters and low zeta potential—a marker for high colloidal stability [45]. Moreover, increasing the pH over 9, might lead to the formation of Ag_2_O black precipitate and thus negatively affecting the SPR peak intensity [27].

### Effect of silver nanoparticle concentration on the absorbance quenching

Different concentrations of PVP-AgNPs solutions ranging from (53.93–215.74 µg/mL) were studied. PVP-AgNPs concentrations above 215.74 µg/mL were excluded as they have absorbance values above 2 units.

### Effect of reaction time on the absorbance quenching

After trying different time intervals for the reaction between TOBRA and PVP-AgNPs, results revealed that the reaction was completed in 5 min, where the absorbance quenching reached its maximum value and remained stable for 30 min. consequently, the reaction time was fixed at 5 min, Supplementary material Fig. S3 shows the reaction time effect on the absorbance quenching.

### Choice of the capping agent

Capping the AgNps with PVP has many advantages over the SDS and citrate which are used as capping agents. The advantages of PVP over the SDS capping agent; 1—the former leads to the synthesis of more uniform particle size, 2—PVP-capped AgNPs have superior antibacterial and anti-fungal activities; which prolongs their shelf-life [46], 3—PVP-capping helps to stabilize the AgNPs by forming steric protective shields, allowing steric and electrostatic stabilization [17], and finally 4—PVP-capping provides excellent physicochemical properties like solubility in both water and organic solvents, biocompatibility, and non-toxicity [47]. Comparing the PVP-capped AgNPs to citrate-capped AgNPs; PVP-capped was found to be more stable [48] and had smaller particle size which improved the method sensitivity [49].

### Optimization of conditions using Quality by Design approach

Quality by Design provides substantial benefits in the field of analytical chemistry, specifically by improving analytical methodologies and saving time and money. A key benefit of QbD is the robustness it brings to analytical processes with the least number of experiments. It guarantees the durability and constant performance of analytical procedures by precisely defining objectives and comprehending the connections between critical procedure factors. Additionally, it improves the predictability, reliability and effectiveness of method development, enabling faster refinement and adjustment of analytical procedures [50, 51].

The full factorial design (2^3^) was used to determine the effect of three factors; the type of buffer, pH and PVP-AgNPs concentration to get the optimum conditions for maximum absorbance quenching for the reaction between TOBRA and PVP-AgNPs with the highest sensitivity. The design permitted to study the significance of each chosen factor and its interaction with the other factors. Two levels for each factor were studied: two types of buffers (Britton-Robinson and carbonate buffers), pH (7 and 9) and PVP-AgNPs concentration (53.93 μg/mL and 215.74 μg/mL), (Supplementary material Table S3). To estimate the model coefficients, ANOVA with linear model was applied. The results of 2^3^ design, are 20 trials, including four center points, (Supplementary material Table S4). The samples were measured by spectrophotometer at 415 nm.

### Analysis of the results

The experimental design results shown in Table 1**,** indicate that the model fits the experimental data as the p-value is < 0.0001 and the F value is equal to 54.73. Buffer type (factor B) and, PVP-AgNPs concentration (factor C) are the most significant factors (with p < 0.0001). The model is linear and revealed that R2 is equal to 0.9746 which means that almost all values of the dependent and independent variables lie on a straight line. Predicted and adjusted R^2^ are equal to 0.8997 and 0.9788, respectively, which indicates that the model is significant and can predict unplanned experiments. Moreover, the adequate precision is greater than 4 which proves that this model is adequate to navigate the space and the difference in results is not random [52].

**Table 1:** ANOVA results of the proposed full factorial model

Source	Sum of squares	Degree of freedom	Mean squares	F value	P values
Model	2.460	7	0.350	54.73	< 0.0001^a^
A-pH	0.170	1	0.170	26.59	0.0004^a^
B-Buffer type	0.370	1	0.370	57.92	< 0.0001^a^
C-PVP-AgNps Concentration	1.290	1	1.290	201.01	< 0.0001^a^
AB	0.072	1	0.072	11.28	0.0073^a^
AC	0.170	1	0.170	25.95	0.0005^a^
BC	0.300	1	0.300	46.05	< 0.0001^a^
ABC	0.048	1	0.048	7.55	0.0206^a^
Pure error	0.064	10	6.414 × 10^–3^		
Cor total	2.560	19			

^a^Significant *p* < 0.05

### The studied factors and their interactions

**Fig. 4:**
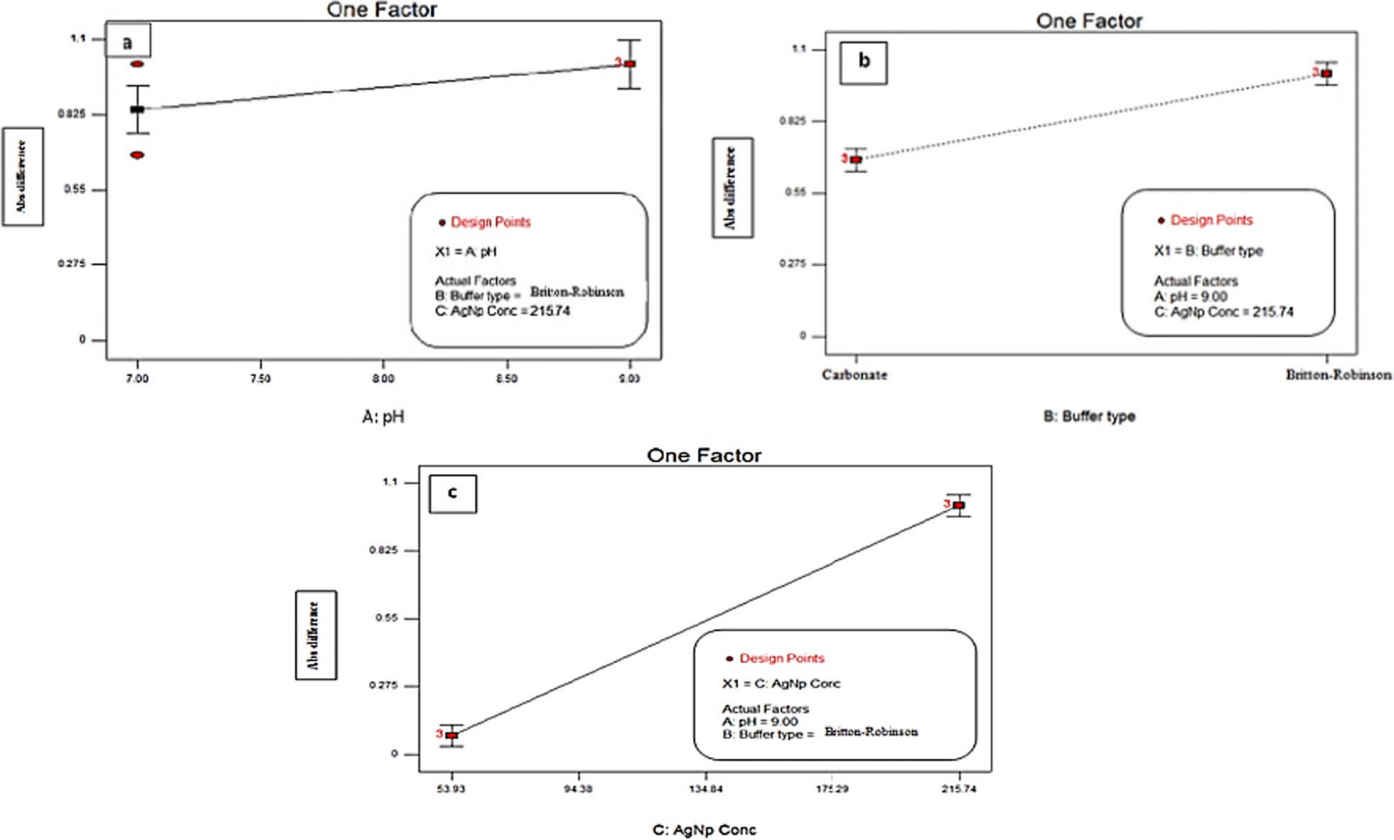
Significance of single factor **a** pH **b** Buffer Type and **c** PVP-AgNPs concentration on the absorbance quenching

The model adequacy checking was applied to explain whether the fitted model provides the approximation to the true system. The significance of one factor plots, (Fig. 4), indicates that the absorbance quenching increases when using Britton-Robinson buffer (factor B), by increasing the PVP-AgNPs concentration (factor C) and by increasing the pH (factor A). (Supplementary material Fig. S4a) showed the interaction between the pH and the buffer while the PVP-AgNPs concentration was kept constant; maximum absorption quenching was achieved at pH 9 with both buffers. (Supplementary material Fig. S4b) showed the interaction between the pH and the PVP-AgNPs concentration using Britton-Robinson buffer; the PVP-AgNPs of concentration (215.74 μg/mL) at pH 9 had higher absorbance quenching than at pH 7 (Supplementary material Fig. S4c) showed the interaction between buffer type and PVP-AgNPs concentration, while the pH was constant; Britton-Robinson buffer showed higher absorbance quenching than Carbonate buffer. The pareto-chart in (Supplementary material Fig. S5) showed the influence of each factor and each interaction on the absorbance quenching; PVP-AgNPs concentration and the buffer type were found to be the most significant factors. The Linear correlation plot of predicted versus actual values showed that almost all the points are close to the 45⁰ diagonal line which means minimum deviation Supplementary material Fig. S6).

**Fig. 5.**
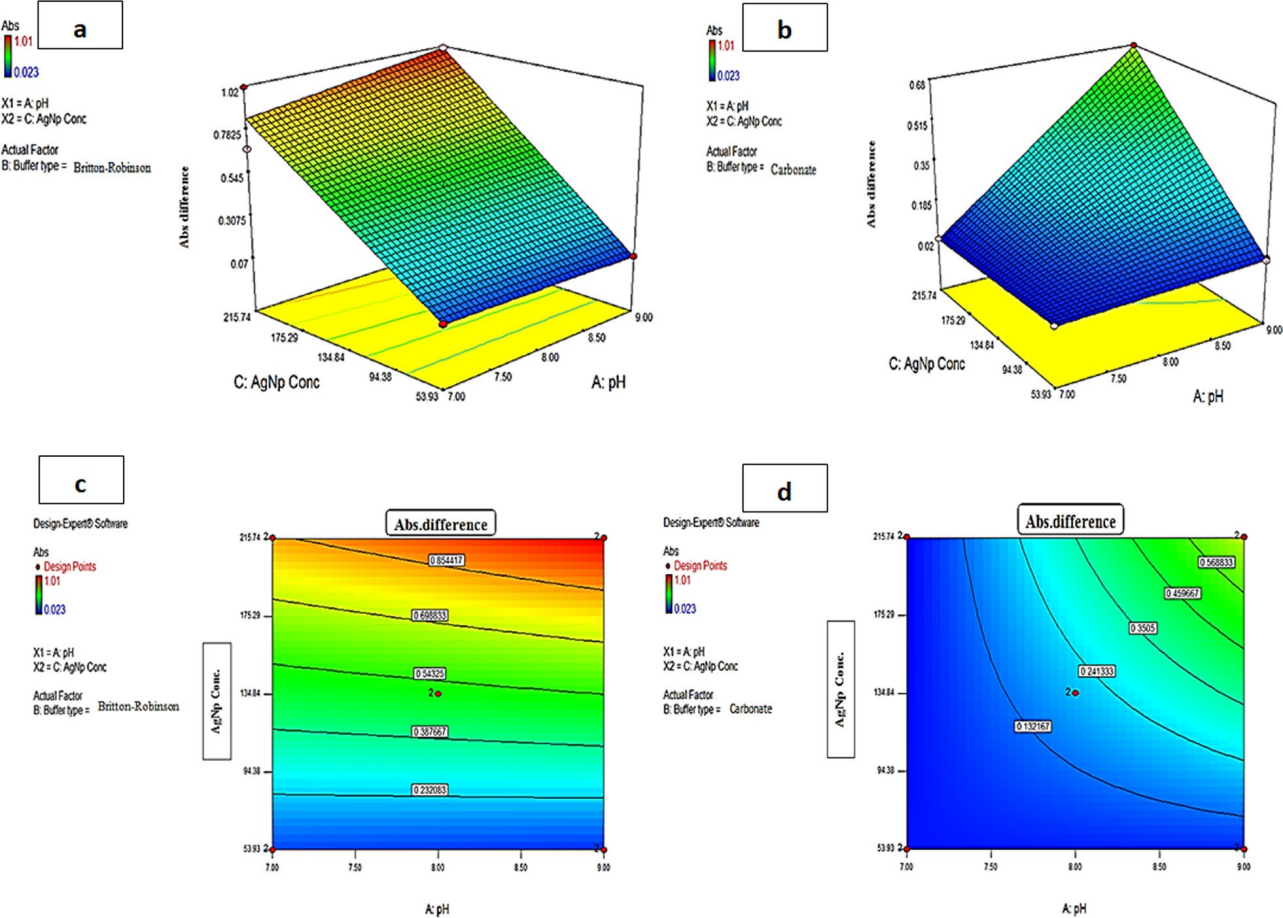
3D-surface plot **a**, **b** and 2D-contour plot **c**, **d** showing the effect of pH and PVP-AgNPs concentration on the absorbance quenching of PVP-AgNPs in presence of 4.00 µg/mL TOBRA in case of using Britton-Robinson buffer **a**, **c** and Carbonate buffer **b**, **d**

The 3D-surface plots (Fig. 5a, b) and two-dimensional (2D) contour plots (Fig. 5c, d) showed the interaction between pH and PVP-AgNPs concentration and its effect on the absorbance quenching value; as the PVP-AgNPs concentration increases, the absorbance quenching increases regardless of the buffer type and pH value. The blue region showed the experiments that resulted in low absorbance quenching and the red region indicated the experiments of high absorbance quenching. We can conclude that the highest absorbance quenching was achieved with PVP-AgNPs concentration range of (175.29–215.74 μg/mL) and Britton-Robinson buffer (Fig. 5c) gave higher absorbance quenching values (red region) than Carbonate buffer (no red region) (Fig. 5d) at the same pH and PVP-AgNPs concentration values. Therefore, the optimum conditions for our method were PVP-AgNPs concentration of 215.74 μg/mL using Britton-Robinson buffer of pH = 9. The validity of the design was proved by the correspondence between the experimental and the predicted results, which was found to be 99.95% at the optimum levels.

### Validation of the proposed method

Calibration curves were constructed by plotting (A⁰–A) versus the corresponding concentration. The regression equation was calculated. The method was linear in the range (0.35–4 μg/mL). The accuracy results were satisfactory expressed by the accepted mean recovery (99.51 ± 0.95). The method was precise intra-daily and inter-daily, confirmed by the RSD values above 2 units. The low values of LOD and LOQ, 0.08 and 0.24 μg/mL respectively, proved the high sensitivity of the method, Table 2.

**Table 2:** Results of assay validation of Tobramycin by the proposed method

Parameters	Results
Linearity (μg/mL)	0.35–4
Slope	0.1903
Intercept	0.3339
Correlation coefficient (r)	0.9997
^ab^Accuracy (mean % ± SD)	99.51 ± 0.95
^ac^Intra-day precision (RSD)	0.871
^ac^Inter-day precision (RSD)	1.384
^d^LOD (μg/mL)	0.08
^d^LOQ (μg/mL)	0.24

^a^Mean of three determinations. ^b^for four concentrations of TOBRA (0.6, 0.7, 2.0 and 2.5 μg/mL). ^c^for three concentrations of TOBRA (1.0, 2.5 and 3.0 μg/mL). Calculated as follow; LOD = 3.3 × Standard deviation of intercept / slope and LOQ = 10 × Standard deviation of intercept / slope

### Application of the proposed method for analysis of pharmaceutical preparations and spiked human plasma

The proposed method was applied for the determination of TOBRA in Tobrin® ophthalmic solution and ointment. The obtained recovery of TOBRA, (Table 3), revealed the absence of excipients interference. The selectivity of the procedure was evaluated by applying the standard addition technique. The ophthalmic route of TOBRA leads to systemic absorption that may cause some side effects such as headache and laryngospasm [53], so the drug plasma concentration needs monitoring. Consequently, the developed method was applied by spiking TOBRA to human plasma samples at three levels, Table 4.

**Table 3:** Determination of Tobramycin in preparations and the application of standard addition technique by the proposed method

Pharmaceutical preparations	*% Found ± SD	Claimed taken: μg/mL	Standard addition technique		
			Pure added μg/mL	Pure Found μg/mL	*% Recovery
Tobrin® Ophthalmic solution BN: 180193	100.54 ± 1.783	0.45	0.25	0.25	99.54
			0.45	0.46	102.22
			1.0	1.00	100.58
			Mean ± SD		100.78 ± 1.35
Tobrin® Ophthalmic ointment BN: 2010036	98.05 ± 0.262	1.00	0.50	0.49	98.72
			1.0	0.99	99.66
			2.0	2.01	100.51
			Mean ± SD		99.63 ± 0.89

*Average of three determinations

**Table 4:** Determination of tobramycin in spiked human plasma by the proposed method

Added (μg/mL)	Found (μg/mL)	*Recovery%
0.50	0.51	102.00%
1.0	1.01	101.00%
5.0	5.1	102.00%
Mean ± SD		101.67 ± 0.577

*Average of three determinations

### Statistical comparison

The statistical comparison of the results obtained by the proposed and colorimetric reported method [4] for the determination of TOBRA is shown in (Table 5). The results showed that there was no significant difference between the two methods in accuracy and precision terms as the calculated t and F values were less than the theoretical ones.

**Table 5:** Statistical comparison between the proposed and the reported methods for determination of Tobramycin

Parameter	Proposed method	Reported method [4]^a^
Mean	99.51	100.74
SD	0.950	0.505
N	4	4
Variance	0.903	0.255
t-test*	2.295 (2.447)	
F*	3.541(15.439)	

^*^Figures in parenthesis are the corresponding theoretical t and F values at (*p* = 0.05)

^a^TOBRA reacts with diphenylamine to form a blue colored complex whose color intensity is measured at 635 nm

### Green assessment of the developed and reported methods

The green character of the developed method was assessed and compared to colorimetric reported methods using Green Analytical Procedure Index (GAPI) and the Analytical GREEnness calculator (AGREE) tools. GAPI tool classifies the greenness of each stage of the analytical method using a scale of three colors representing the degree of environmental impact [28, 54]. For our developed method, the green color dominates. There are four yellow segments (1, 9, 10 and 11) and four red segments (5, 6, 7 and 14). The green character of the two reported methods for the determination of TOBRA using SDS-AgNPs [23] and using diphenylamine [4] was compared to the developed one. The Reported method [23] has six red segments due to the extraction step of milk samples, utilization of a large volume of solvents and consequently producing a large amount of waste, which was not treated. Reported method [4] has seven red segments due to the same causes as the first reported method in addition to using the derivatization step, which is not considered green treatment. The assessment results of GAPI are listed in Table 6 showing that the developed method is greener than the two reported methods. The newly launched Analytical GREEnness calculator (AGREE) allows automatic, rapid, and informative assessment depending on the 12 principles of green analytical chemistry (SIGNIFICANCE) [29]. According to AGREE pictograms, shown in Table 6, the final score of the developed method is higher than the other two reported methods with a value of 0.74, which is mainly due to the larger amount of solvent used and the presence of the derivatization step in reported method [4] which is not considered a green step. In conclusion, the proposed method has a higher green profile than the reported methods. A graphical representation of the greenness study is shown in (Supplementary material Fig. S7).

**Table 6:** Greenness assessment of the proposed and reported methods for determination Tobramycin by GAPI and AGREE tools

Category	Proposed method	Reported method [23]	Reported method [4]
Sample collection [1]	At-line	At-line	At-line
Sample preservation [2]	None	None	None
Sample transport [3]	None	None	None
Sample storage [4]	None	None	None
Type of method: direct or indirect [5]	Indirect	Indirect	Indirect
Scale of extraction [6]	Macro-extraction	Macro-extraction	Macro-extraction
Solvents/reagents used [7]	Non-green solvents	Non-green solvents	Non-green solvents
Additional treatments [8]	None	None	Derivatization required
Amount [9]	10–100 mL	> 100 mL	> 100 mL
Health hazard [10]	Acetic acid: NFPA health hazard = 3 Chloroform: NFPA health hazard = 2 AgNps: NFPA health hazard = 0	SDS: NFPA health hazard = 2 Chloroform: NFPA health hazard = 2	Acetic acid: NFPA health hazard = 3 Diphenylamine: NFPA health hazard = 2 Hydrochloric acid: NFPA health hazard = 3
Safety hazard [11]	Acetic acid: flammability = 2 Chloroform: flammability = 0 AgNps: flammability = 0	SDS flammability = 3 Chloroform: flammability = 0	Acetic acid: flammability = 2 Diphenylamine: flammability = 1 Hydrochloric acid: flammability = 0
Energy [12]	≤ 0.1 kWh/ sample	≤ 0.1 kWh/ sample	≤ 0.1 kWh/ sample
Occupational hazard [13]	Hermetic sealing of analytical process		
Waste [14]	> 10mL	> 10mL	> 10mL
Waste treatment (15)	Recycling	None	None
Quantification	Yes	Yes	Yes
GAPI			
AGREE			

## Conclusion

In this work, an eco-friendly, simple, rapid, and sensitive optical sensor was developed and validated for the determination of non-UV-absorbing drug TOBRA using PVP-AgNPs. The optimization of the reaction conditions to get maximum absorbance quenching was done using a factorial design by studying three factors: PVP-AgNPs concentration, pH, and buffer type. PVP-AgNPs concentration of (215.74 μg/mL) and the use of Britton-Robinson buffer of pH 9 gave the most significant effect. The proposed method was linear in the range of (0.35–4 μg/mL). The method is sensitive with LOD = 0.08 μg/mL and permits rapid analysis of TOBRA without the need for derivatization nor sample pre-treatment. The method is greener compared to the reported colorimetric methods according to GAPI and AGREE tools. The interaction between the drug and PVP-AgNPs was assessed by the molecular docking software. Finally, the optical sensor allows the determination of TOBRA in different pharmaceutical dosage forms (eye solution and ointment) and spiked human plasma with good recovery. Being green, simple, non-expensive, rapid, and utilizing the spectrophotometer, which is available in every lab, it could be used for the quality control tests of TOBRA in bulk powder and pharmaceutical formulations.

### Supplementary Information

Spplementary Material 1.

## Data Availability

All data generated or analyzed during this study are included in this published article.

## Abbreviations

AGREE: Analytical GREEnness calculator
Ag-NPs: Silver nanoparticles
ATR: Attenuated total reflectance
EBC: Exhaled breath condensate
GAPI: Green Analytical Procedure Index
LC: Liquid chromatography
PVP: Polyvinyl pyrrolidone
SDS: Sodium dodecyl sulfate
SPR: Surface Plasmon resonance
TEM: Transmission electron microscopy
TOBRA: Tobramycin
