# Supplementary material for: Implementation of green-assessed nanotechnology and quality by design approach for development of optical sensor for determination of tobramycin in ophthalmic formulations and spiked human plasma
Source: BMC Chem. 2024 Jul 15;18(1):131. doi: 10.1186/s13065-024-01234-y (PMC11247747; doi:10.1186/s13065-024-01234-y)
Supplement: Supplementary file 1 — Spplementary Material 1. [file 13065_2024_1234_MOESM1_ESM.docx]

**Supplementary material**

**
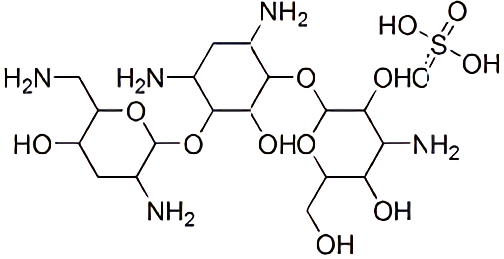
**

**Fig.S1.** Chemical structure of Tobramycin sulfate


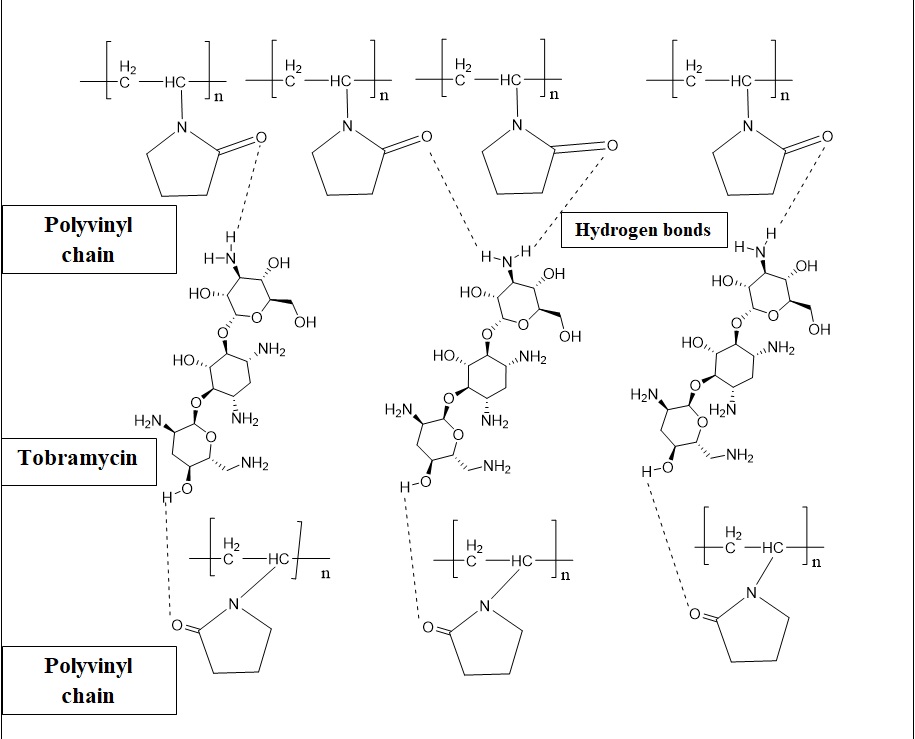


Fig.S2. A diagram showing the binding of PVP-AgNps with Tobramycin

**Fig.S3.** The effect of reaction time on the absorbance quenching of SPR peak of PVP-AgNPs,

using 4μg/ml tobramycin sulfate and Britton-Robinson buffer pH 9.


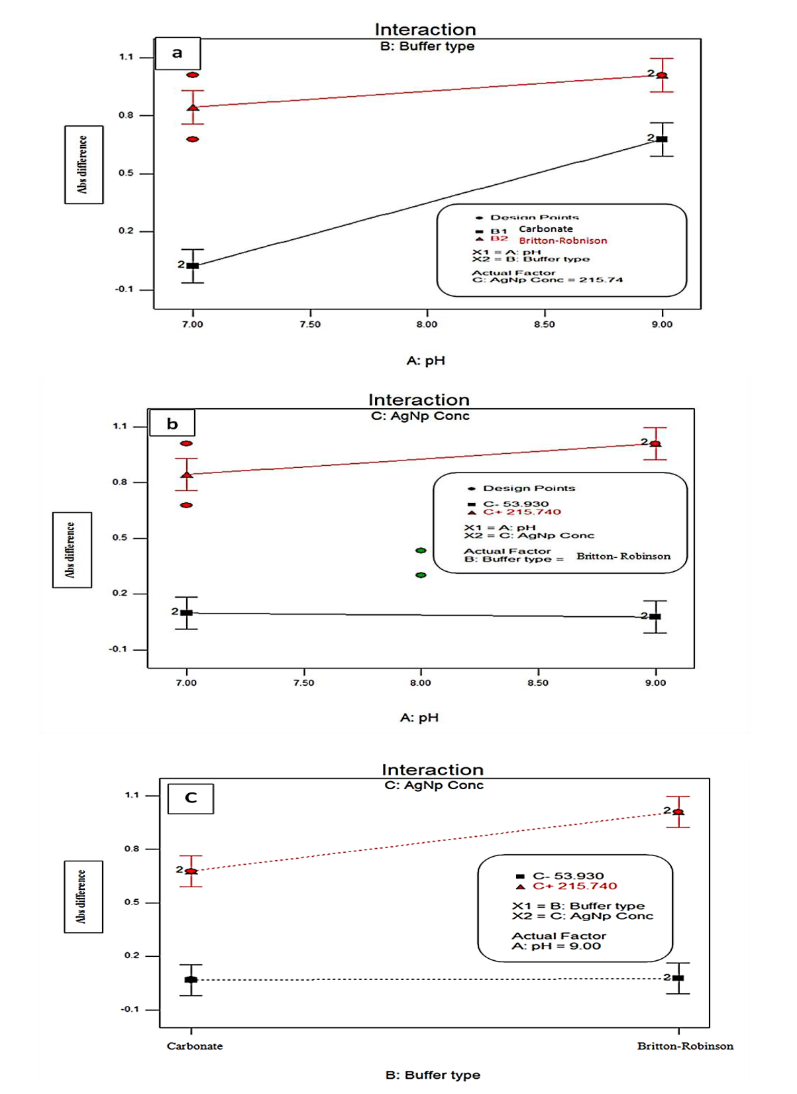


**Fig.S4.** The interaction between **(a)** pH and buffer type **(b)** pH and PVP-AgNPs concentration,

**(c)** Buffer type and PVP-AgNPs concentration while the third factor was kept constant.

**
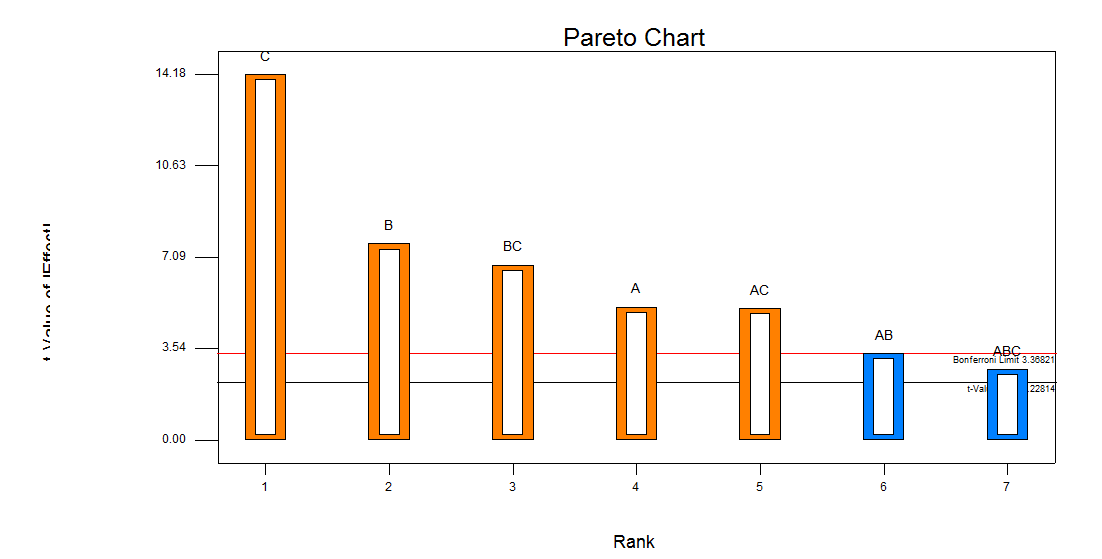
**

**A:** pH **AB:** Interaction between pH and buffer type

**B:** Buffer Type **BC:** Interaction between Buffer type and PVP-AgNps conc

**C:** PVP-AgNpsconc. **AC:** Interaction between pH and PVP-AgNpsconc.

**ABC:** Interaction between pH, buffer type and PVP-AgNps conc.

**Value of effect**

**Fig.S5.** Pareto graph to show the influence of variables on absorbance quenching


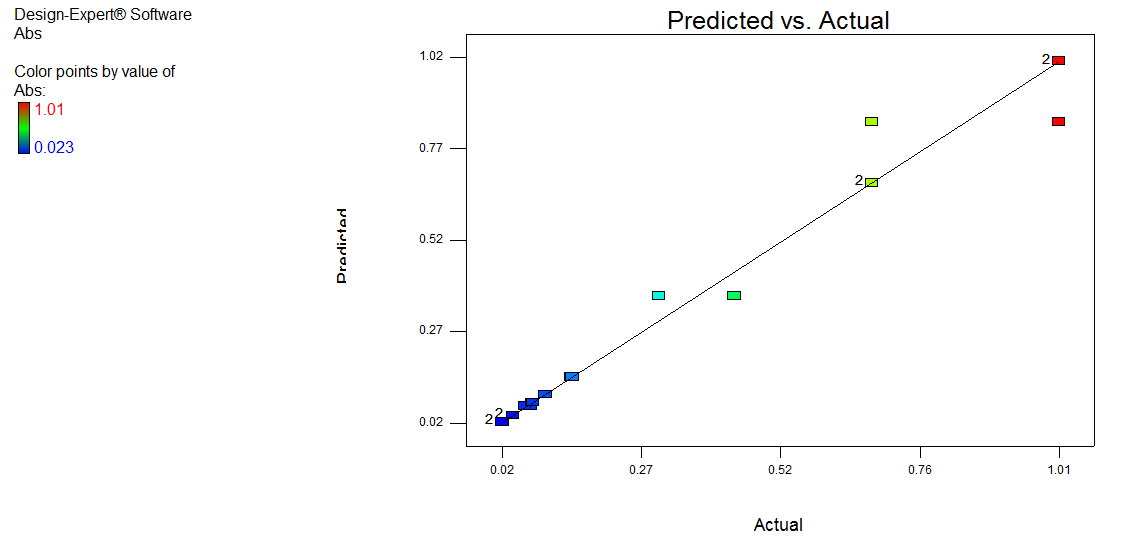


**Predicted**

**Fig.S6.** Linear correlation plot between the actual and the predicted values

**
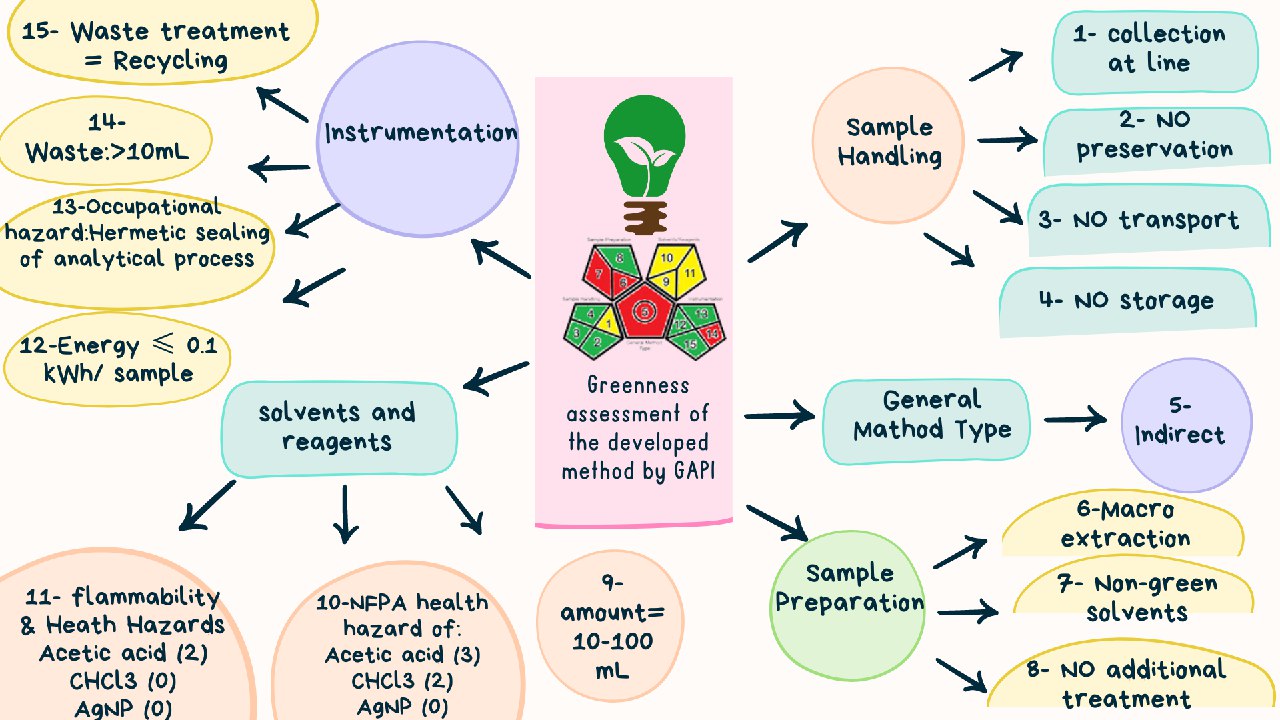
**

**Fig.S7.** A graphical representation for the greenness study using GAPI tool

**Table S1.** Table of comparison between the spectrophotometric/colorimetric methods and the proposed method for tobramycin determination using capped silver nanoparticles

| **Point of comparison** | **Reported method [23]** | **Reported method [24]** | **Reported method [25]** | **Reported method [26]** | **Proposed Method** |
| --- | --- | --- | --- | --- | --- |
| **Type of Capping or stabilizer** | Sodium dodecyl sulfate (SDS) | Sodium dodecyl sulfate (SDS) | Sodium dodecyl sulfate (SDS) | Citrate  PolyA aptamer in presence of poly diallyl dimethyl ammonium chloride (PDDA) | Polyvinyl pyrrolidone (PVP) |
| **Mechanism of Interaction** | Interaction of amine groups amine groups and AgNPs  results in the formation of strongly adsorbed drug on the surface of AgNPs which results in decrease in SPR  band of AgNPs. | Interaction of sodium metaborate(serving as the linker ) with the SDS-capped AgNPs and tobramycin results in aggregation of the nanoparticles  and consequently decreases the absorbance intensity. | NA | Aggregation induced by tobramycin binding to aptamer-coated AgNPs upon Tobramycin addition, aptamer bind to it which leads to release of PDDA and subsequent aggregation of AgNPs leading to change of colorimetric analytics signal. | The mechanism of PVP-AgNPs aggregation is due to hydrogen bond formation between the lone pair of electrons of the oxygen in the polyvinyl groups of the PVP-AgNPs and hydroxyl and amino groups of Tobramycin. |
| **Linear Range (ng/mL or µM)** | 22.5–60 ng/mL | 1.0–50.0 ng/mL | NA | 0.1–100 nM | 350–4000 ng/mL |
| **LOD (ng/mL or nM)** | 5 ng/mL | 0.5 ng/mL | NA | 0.07 nM | 80 ng/mL |
| **LOQ (ng/mL)** | 14 ng/mL | NA | NA | NA | 240 ng/mL |
| **Precision (% RSD)** | ≤5% | Intra-day: 2.8%,  Inter-day: 4.2% | NA | NA | Intra-day: 0.871%  Inter-day: 1.384 |
| **Use of Quality by Design** | NA | Yes, response surface method (RSM) used | NA | NA | Yes, Full factorial design |
| **Greenness Assessment** | NA | NA | NA | NA | Yes, using GAPI and AGREE tools |
| **Type of Matrix for Application and Practicality in Real World** | Milk and cream formulations, not injections containing EDTA | Exhaled breath condensate (EBC) | Exhaled breath condensate (EBC) | Milk | Spiked Human Plasma |
| **Robustness (parameters varied)** | pH, NaCl concentration, and  measurement time. | The robustness of the method for the determination of tobramycin in EBC samples is not explicitly mentioned. However, RSM was employed to study the influence of various reaction parameters, including pH, reagent concentrations, and reaction time, which could impact the tobramycin analysis | NA | NA | A key benefit of Quality by Design is the robustness it brings to analytical processes.  Full factorial design was employed to study the effect of different reaction parameters, including concentration of PVP-AgNps ,pH, and type of buffer used, which could impact the tobramycin analysis |

**Table S2.** Table of comparison between the two published colorimetric methods and the proposed method for tobramycin determination

| **Point of comparison** | **Diphenylamine Method [4]** | **Vanillin Method [5]** | **Proposed Method** |
| --- | --- | --- | --- |
| **Linear Range (µg/mL)** | 0.5 - 3 | 3 - 15 | 0.35- 4 |
| **LOD (µg/mL)** | NA (Lower limit of quantification given as 0.5 µg/mL) | NA (Not specifically mentioned) | 0.08 |
| **LOQ (µg/mL)** | 0.5 | NA (Not specifically mentioned) | 0.24 |
| **Precision (RSD %)** | < 2% | < 2% (Relative standard deviations from quantitative analysis) | < 2% |
| **Use of Quality by Design** | Not mentioned | Not mentioned | QBD was applied |
| **Greenness Assessment** | Not mentioned | Not mentioned | Greenness was assessed |
| **Presence of Derivatization Step** | Yes, forms a colored complex with diphenylamine under drastic conditions | Yes, involves the formation of a  Schiff's base with vanillin | No derivatization |
| **Type of Matrix for Application** | Eye drops, ointment | Parenteral and ophthalmic preparations | Pharmaceutical formulation ( solution and ointment ) and spiked human plasma |
| **Robustness** | Evaluated through change of DPA volume and reaction time ; method insensitive to small variations in experimental parameters | Not explicitly mentioned, but derivative  was stable up to 72 hours | A key benefit of Quality by Design is the robustness it brings to analytical processes.  Full factorial design was employed to study the effect of different reaction parameters, including concentration of PVP-AgNps, pH, and type of buffer used, which could impact the tobramycin analysis. |
| **Practicality in Real World** | Practical for bulk and pharmaceutical formulations | Practical for pharmaceutical preparations | Being green, simple, non-expensive, rapid, and utilizing the spectrophotometer, which is available in every lab, it could be used for the quality control tests of TOBRA in bulk powder and pharmaceutical formulation |

**Table S3.** The three variables at two levels

| **Variable** | **Level (-1)** | **Level (+1)** |
| --- | --- | --- |
| **pH** | 7 | 9 |
| **PVP-AgNPsConcentration** | 53.93 μg/mL | 215.74 μg/mL |
| **Buffer Type** | Carbonate buffer | Britton-Robinson buffer |

**Table S4.** The 2^3^ full factorial design showing the used levels of factors.

| Run | pH | Buffer Type | PVP-AgNPs concentration  (μg/mL ) | Absorbance quenching |
| --- | --- | --- | --- | --- |
| 1 | 9 | Britton-Robinson | 215.74 | 1.01 |
| 2 | 8 | Carbonate | 134.84 | 0.147 |
| 3 | 8 | Britton-Robinson | 134.845 | 0.3 |
| 4 | 7 | Carbonate | 53.93 | 0.041 |
| 5 | 7 | Carbonate | 53.93 | 0.041 |
| 6 | 9 | Britton-Robinson | 215.74 | 1.01 |
| 7 | 9 | Carbonate | 53.93 | 0.063 |
| 8 | 7 | Britton-Robinson | 215.74 | 1.01 |
| 9 | 7 | Carbonate | 215.74 | 0.023 |
| 10 | 7 | Britton-Robinson | 53.93 | 0.098 |
| 11 | 7 | Britton-Robinson | 53.93 | 0.099 |
| 12 | 9 | Carbonate | 53.93 | 0.073 |
| 13 | 9 | Britton-Robinson | 53.93 | 0.076 |
| 14 | 8 | Carbonate | 134.84 | 0.145 |
| 15 | 7 | Carbonate | 215.74 | 0.023 |
| 16 | 8 | Britton-Robinson | 134.84 | 0.434 |
| 17 | 7 | Britton-Robinson | 215.74 | 0.678 |
| 18 | 9 | Britton-Robinson | 53.93 | 0.077 |
| 19 | 9 | Carbonate | 215.74 | 0.678 |
| 20 | 9 | Carbonate | 215.74 | 0.678 |
